# Transactional sex among adolescent girls and young women enrolled in a cash plus intervention in rural Tanzania: a mixed‐methods study

**DOI:** 10.1002/jia2.26038

**Published:** 2022-11-30

**Authors:** Meghna Ranganathan, Sarah Quinones, Tia Palermo, Ulrike Gilbert, Lusajo Kajula, Tanzania Cash Plus Evaluation Team, Tia Palermo, Sarah Quinones, Lusajo Kajula, Jacobus de Hoop, Leah Prencipe, Valeria Groppo, Nyasha Tirivayi, Jennifer Waidler, Johanna Choumert Nkolo, Respichius Mitti, Marie Mallet, Bhoke Munanka, Paul Luchemba, Tumpe Mnyawami Lukongo, Aroldia Mulokozi, Ulrike Gilbert, Paul Quarles van Ufford, Rikke Le Kirkegaard, Frank Eetaama, Jennifer Matafu, Diego Angemi, Luisa Natali

**Affiliations:** ^1^ London School of Hygiene and Tropical Medicine London UK; ^2^ Department of Epidemiology and Environmental Health University at Buffalo Buffalo New York USA; ^3^ HIV and AIDS Unit UNICEF Tanzania Dar es Salaam Tanzania; ^4^ UNICEF Office of Research—Innocenti Dar es Salaam Tanzania

**Keywords:** adolescent girls and young women, cash plus, HIV risk, social protection, Tanzania, transactional sex

## Abstract

**Introduction:**

Transactional sex or material exchange for sex is associated with HIV infection among adolescent girls and young women in sub‐Saharan Africa. The motivations for engaging in transactional sex vary from the fulfilment of basic needs, to enhancing social status or for romantic reasons with the expectation that men should provide. Transactional sex is also associated with HIV risk behaviours, such as multiple sexual partners and other determinants of HIV risk, including partner violence and abuse, alcohol consumption and inconsistent condom use.

**Methods:**

We use data from a mixed‐method, cluster randomised controlled trial of the *Ujana Salama* cash “plus” intervention in rural Tanzania. The data are from the first and third rounds of data collection (2017–2019). The impact evaluation consisted of a parallel mixed‐methods design where the quantitative and qualitative data collection occurred simultaneously, and integration of the findings was done during the discussion. We first examine contextual factors associated with transactional sex using multivariable logistic regression models and then estimate whether the “plus” intervention reduced transactional sex among adolescent girls and young women using analysis of covariance. We used thematic content analysis for analysing qualitative transcripts.

**Results:**

The prevalence of transactional sex among unmarried adolescent girls and young women at round 3 was 26%. Findings show that increasing age is a risk factor for transactional sex (OR = 1.80; 95% CI: [1.50, 2.17]), staying in school was negatively associated with engagement in transactional sex (OR = 0.24; 95% CI: [0.14, 0.40]). The cash plus intervention showed no impacts on reducing transactional sex (β = 0.003, *p* = 0.905).

**Conclusions:**

The mechanisms of impact for a cash plus intervention on transactional sex are complex; economic insecurity is an important driver of transactional sex and HIV infection, but psychosocial factors and gendered social norms need consideration in intervention development. Our findings suggest that combination prevention interventions to address the structural drivers of HIV infection should focus on efforts to increase school enrolment and completion.

## 1 INTRODUCTION

Adolescence is a developmental period involving significant physical, cognitive and psychological changes that mark the transition to adulthood, including shifts in social and gender roles and responsibilities [1, 2]. Along with social and economic pressures accompanying this transition to adulthood, adolescents can become particularly vulnerable to HIV infection, as they become sexually active or begin to experiment with drugs and alcohol [3]. While there is an overall decrease in HIV incidence in sub‐Saharan Africa (SSA), adolescent girls and young women (AGYW) still remain at increased risk and account for 25% of new infections [4]. In Tanzania, HIV prevalence is 1.3% among adolescent girls aged 15–19 years and 4.4% among young women aged 20–24 years. Structural factors, such as social and economic inequalities, household poverty and food insecurity, poor quality education and gender‐based violence limit opportunities for AGYW [5]. Where HIV prevalence is high, these factors combine with increased biological vulnerability and limited knowledge about HIV to render AGYW more vulnerable to HIV infection [3, 5].

Transactional sex is defined as “non‐marital, non‐commercial sexual relationships motivated by the implicit assumption that sex will be exchanged for material benefit or status” [6]. Evidence has demonstrated that AGYW who report transactional sex are at a higher risk of HIV acquisition [6, 7, 8, 9, 10]. Transactional sex is also associated with risky sexual behaviours, such as multiple sexual partners [11, 12], and other determinants of HIV risk, including partner violence [11, 13], alcohol consumption [14, 15] and varying levels of condom use [16, 17]. However, not all forms of transactional sex are risky for HIV infection. Qualitative evidence suggests that transactional sex is an expectation embedded in adolescent romantic relationships; and certain aspects related to male provision or dependence on partners for money or material support result in young women's weakened negotiating position within the relationship that may make it risky for HIV infection [18].

The motivations for AGYW's engagement in transactional sex are varied. They may resort to it for basic needs when their economic situation is precarious or their livelihood opportunities are constrained [17]. They may also engage in transactional sex to increase their social status or may engage in relationships that are predicated on love where the receipt of money and sex are intertwined [18, 19]. However, as not all sexual relationships characterised by or involving exchange are inherently risky, and motivations for engaging in transactional sex are complex, the emphasis should be placed not on eliminating transactional sex but rather on identifying the conditions and circumstances in which transactional sex imparts HIV risk [20].

Cash transfers are increasingly popular in SSA to reduce poverty, improve food security and increase productive activities [21]. There is evidence that cash transfers have delayed the age of first sex and pregnancy [22, 23, 24], reduced transactional sex and age‐disparate sex, reduced intimate partner violence [25, 26] and increased school enrolment [27]; however, programmatic effects have varied by context and implementation and design features and these protective effects have not been replicated in all countries where examined. To this effect, there is recognition that cash alone may not be sufficient to reduce the interrelated social, psychological and economic risks faced by AGYW.

Cash “plus” interventions combine cash transfers with one or more types of complementary support [28], such as behaviour change communication, psychosocial support or linkages to services. These bundled programmes have the potential to facilitate safe transitions to adulthood and reduce HIV risk for AGYW [29, 30]. For instance, in Zambia, the Adolescent Girls Empowerment Programme combined mentoring and safe spaces with health, life skills and financial education, and a savings account. The programme found positive impacts on outcomes, such as having a safe place to meet with friends, financial literacy, a reduction in reported transactional sex and increased condom use at first sex, but did not have impacts on educational attainment, delays in pregnancy and marriage, the experience of violence or HIV/herpes simplex virus‐2 infection [24]. Qualitative evidence from a non‐governmental organization (NGO)‐implemented cash plus intervention (providing cash combined with financial education) targeted to AGYW in Tanzania showed that the intervention reduced dependence on male sex partners for basic needs [31]. Given mixed findings on sexual and reproductive health (SRH) that varies by context and design, there is a need to explore the impacts of a cash plus programme on AGYW SRH, including engagement in transactional sex.

In the current study, we evaluate the impacts of the “plus” components from a cash plus intervention for adolescents, implemented within the platform of a national social protection programme in Tanzania. The intervention was developed to target outcomes across various domains of wellbeing related to safe transitions to adulthood, including transactional sex. We examine both the contextual factors associated with transactional sex in rural Tanzania and whether the “plus” intervention reduced engagement in transactional sex.

## 2 METHODS

The data for this analysis are drawn from rounds 1 and 3 of the impact evaluation (2017–2019) of the “*Ujana Salama*” Cash Plus model for safe transitions to a healthy and productive adulthood impact evaluation (*details below*). This is a cluster randomised controlled trial (RCT) with a parallel mixed‐ methods design, where the quantitative survey and qualitative data collection occurred simultaneously [32]. We integrated the findings from the survey data and qualitative interviews when interpreting the results and triangulate the findings in the discussion.

### 2.1 Study design and details: Tanzania's Productive Social Safety Net and the “plus” components (“Ujana Salama”)

The United Republic of Tanzania's Productive Social Safety Net (PSSN) programme (i.e. the “cash”) is implemented by the Tanzania Social Action Fund (TASAF), a government agency. The objectives are to increase income and consumption, improve vulnerable populations’ ability to cope with shocks, invest in human capital and increase access to improved social services. By 2019 (when the PSSN Phase 1 ended and data were last collected for the current study), the programme reached one million households nationally. PSSN benefits comprise (1) a bi‐monthly cash transfer; (2) a public works programme during the lean season; and (3) a livelihood enhancement component. The cash transfer is comprised of a basic fixed (unconditional transfer), and an additional cash transfer conditional on “co‐responsibilities” related to health seeking (for young children and the elderly) and children's school enrolment. The targeting is conducted in a four‐stage process: (1) geographical targeting to identify and select districts, wards and villages; (2) community targeting to identify extremely poor and vulnerable households in selected villages; (3) a proxy means test (PMT) to verify and minimize inclusion errors among non‐poor households; and (4) a community validation test to confirm the results of the community targeting and PMT.

The title of the “plus” intervention evaluated in the current study is “*Ujana Salama*” and was implemented between January 2018 and July 2019 by TASAF, with technical assistance from UNICEF and the Tanzania Commission for AIDS (TACAIDS). The intervention aimed to facilitate safe transitions to adulthood, simultaneously promoting economic strengthening (e.g. starting a business and engagement in productive activities), health capabilities (e.g. knowledge, HIV testing and access to health facilities) and protection outcomes (reductions in violence and exploitation proxied by engagement in risky forms of transactional sex). The Ujana Salama pilot was randomised among 130 villages in four districts/councils (Mufindi and Mafinga in Iringa region and Busokelo and Rungwe in Mbeya region) to rigorous evaluation. In the treatment villages (*n* = 65), the plus component was targeted to all adolescents aged 14–19 years (at survey baseline in 2017) living in PSSN households. In the control villages, households continued to receive the PSSN but did not receive the Ujana Salama “plus” intervention activities, which included (1) livelihood and SRH life skills training; (2) mentoring and asset transfer (80 United States dollars); and (3) supply‐side strengthening of adolescent‐friendly HIV and SRH services and linkages to existing SRH and HIV services for adolescents.

A timeline of activities is provided in Figure S1, and topics covered in the training curriculum are provided in Table S1. Trainings were led by facilitators who were trusted and trained adults from the local community. They met with the participants once, for 2 hours, per week during the training period (January–May 2018). During the mentoring phase (June 2018–March 2019), mentors provided guidance to adolescents on livelihood options and healthy life choices. During this phase, adolescents developed an education or business plan, and upon approval of the plans, productive grants were disbursed to the adolescents via the PSSN payment structures (in April and July 2019). The supply‐side strengthening activities took place in July 2018, when the Ministry of Health, Community Development, Gender, Elderly and Children conducted trainings to strengthen the provision of adolescent‐friendly SRH and HIV services in government‐run primary health facilities in the study areas.

### 2.2 Quantitative design and analysis

The impact evaluation was led by UNICEF Office of Research—Innocenti, in collaboration with EDI Global, University of Buffalo, United States of America (USA), UNICEF Tanzania and Government partners (TASAF and TACAIDS). Detailed information on the evaluation can be found elsewhere [33]. Briefly, it examined the influence of adolescent‐focused intervention components on adolescent (ages 14–19) wellbeing. These adolescents were living in impoverished rural Tanzanian households that received the PSSN programme. For the impact evaluation, 130 villages in the two districts were randomised into treatment or control arms at a 1:1 ratio, using a design stratified by district and village size (large vs. small villages). Randomisation occurred after baseline surveys were implemented. The three rounds of data collection for this impact evaluation were conducted by EDI Global between April 2017 and August 2019 (see study timeline in Figure S1). Data used in the current study come from round 1 (2017) and round 3 (2019).

In selected villages (treatment and control), all adolescents in PSSN households were targeted for interviews. Eligibility criteria for interviewed youth in this study include (1) living in a PSSN household and (2) being 14–19 years of age at baseline data collection (May–June 2017). Youth interviews were conducted in a private location within the youth's household by same‐sex enumerators in Swahili, and responses were entered into computer‐assisted personal interview software. Topics covered in adolescent interviews included time‐use, livelihoods skills and knowledge, economic activity participation, sexual activity/risk‐taking behaviours, pregnancy, marriage, education, aspirations, psychosocial wellbeing and violence victimisation. Violence modules were administered to half of the eligible sample. Household surveys were conducted with the head of the household where the adolescent resided to assess household conditions. Additionally, community questionnaires were administered to a group of individuals with adequate community knowledge that include questions about access to markets, health facilities and schools; prices; marriage customs; caregiving; and community shocks.

### 2.3 Measures

#### 2.3.1 Outcome variable

The outcome of interest for this analysis is engagement in transactional sex among females who were (1) unmarried at round 3; and (2) surveyed at both baseline and round 3 irrespective of sexual debut (transactional sex was coded as = 0 if reported no sexual debut). Guided by previous research on transactional sex by the Tackling the Structural Drivers of HIV (STRIVE) consortium transactional sex working group [34], Stoebenau et al. [6], Wamoyi et al. [35], Ranganathan et al. [8], as well as drawing on questions recommended by Wamoyi et al. [8, 36], we created an additive transactional sex index from the responses to the following questions: (1) Would you leave the relationship if [recent partner] did not give you money or things that are important to you? (2) Has [most recent partner] ever given you money? (3a) What are the three main reasons you are/were with [most recent partner]? (3b) In the past 12 months, did you start a sexual relationship with [recent partner] in order to get things that you needed, such as money or gifts? Affirmative responses to questions 1 or 2 were coded as = = 1 and 0 otherwise. For question 3a, if responses included money, gifts or assistance, they were coded as = = 1 and 0 otherwise and for 3b, affirmative responses were coded = = 1 and 0 otherwise to generate an additive transactional sex scale range of 0–3. Further, for the purposes of this analysis, the additive scale was dichotomised and those who reported at least one of the above were = = 1 and coded 0 otherwise.

#### 2.3.2 Covariates and potential confounders

The variables considered in this analysis include individual level (age, locus of control, self‐esteem, quality of life, current school attendance or completion of Form IV, number of sexual partners in the past 12 months and experiences of sexual violence in the past 12 months); household level (household size, living in a female‐headed household and age of household head); and community‐level indicators (distance to market, distance to secondary school and gender‐equitable norms in the community) (please see Table S2).

### 2.4 Statistical analyses

Descriptive statistics are provided for the panel sample of AGYW who participated in the baseline and round 3 surveys of the impact evaluation. Statistics include means accompanied by standard deviations (SD) and percentages of individual, household and community‐level characteristics presented by engagement in transactional sex. Bivariate logistic regressions were used to calculate odds ratios (OR) and 95% confidence intervals (CI) to estimate the crude associations between sample characteristics and transactional sex experiences at round 3.

Next, we implemented multivariable logistic regression models to calculate the OR and 95% CI to estimate the association between contextual factors and transactional sex. We present two adjusted models: one including sexual violence (model 1) and one excluding sexual violence (model 2). The choice of two models was based on the loss of sample size in the violence model given the split‐sample approach. The multivariable logistic regression models included adjustments for the age of the household head, living in a female‐headed household and district of residence × size of district (definitions in Table S2). The multicollinearity of the explanatory variables in the final logistic regression model was tested and variables with a variance inflation factor greater than two were excluded.

Finally, we performed an analysis of covariance (ANCOVA) to estimate programme impacts and examine whether the PSSN Cash plus programme mitigated the risk of transactional sex experiences, using the dichotomized transactional sex variable as our outcome. The ANCOVA model is a linear regression model that controls for adolescents’ reports of transactional sex at baseline, district of residence and district size, and baseline age. Standard errors were adjusted for clustering at the village level.

### 2.5 Qualitative design and analysis

To better explore themes and pathways of impact, qualitative interviews were conducted with a subset of participants (40 baseline and 32 round 3) from the quantitative sample. The sub‐sample at baseline consisted of male and female adolescents aged 14–19 from the Southern Highlands of Tanzania. Interviews were conducted primarily with the same youth at 12‐month intervals. To improve retention, in a few cases where previously interviewed youth could not be found, replacement interviews were conducted with youth from PSSN household who fit the criteria. Same‐sex interviews were conducted in Swahili. Interviews were digitally recorded, transcribed verbatim into Swahili, and then translated into English. Adolescents and youth were interviewed about their romantic relationships, their reasons for starting romantic relationships, whether they started relationships based on material exchange and whether they would stop dating someone if he/she would not give them gifts or money. We also explored the dynamics of poverty and transactional sex among these youth.

Qualitative data collection and analysis utilised an iterative approach that included adjusting the interview guides to explore emergent themes. Analysis was conducted in two phases: (1) rapid initial analysis to document observations during fieldwork and (2) in‐depth analysis. Codes were created using a priori themes from the interview guides and were supplemented with themes that emerged during data analysis [37, 38]. Data were coded using the MAXQDA software program (MAXQDA 11—Software for Qualitative Data Analysis 1989–2016. Berlin, Germany: VERBI). A team of three was assigned a subset of the transcripts to code using the codebook, while 12 transcripts were coded by all to assess intercoder reliability. Whenever coding discrepancies occurred, the coders discussed and reached an agreement. Also, themes that did not appear to fit the existing codes were discussed and considered for recoding.

### 2.6 Ethical approvals

Ethics approval for the study was granted by the National Institute for Medical Research (NIMR/HQ/R.8c/Vol.I/1233) and permission to conduct the study was granted by the Tanzania Commission for Science and Technology (COSTECH). Informed consent was obtained from all individuals aged 18 and above years, as well as married adolescents of any age, and caregiver/parental consent and youth assent was obtained for all unmarried adolescents aged 14–17 years. For modules on violence victimisation, a split sample approach was used, meaning that violence modules were alternately administered in one village for females and a second village for males. This approach serves to protect the safety and confidentiality of respondents, eliminating the chance that a male perpetrator and a female victim living in the same community are both interviewed, and follows similar practices implemented by Violence Against Children Surveys previously implemented in Tanzania [39].

## 3 RESULTS

### 3.1 Sample characteristics

The prevalence of reported transactional sex at round 3 among unmarried AGYW was 26% (221/864). AGYW who reported engaging in transactional sex were, on average, older (16.9 years) than those who did not (15.4 years; *p*<0.001), and a higher proportion came from a household where the head had no formal education relative to AGYW who had not engaged in transactional sex (*p* = 0.048; see Table 1). Adolescents who did not engage in transactional sex were marginally higher on the self‐esteem scale compared to those who did engage in transactional sex (3.97 vs. 3.86; *p* = 0.061). Nine percent of adolescents who reported engaging in transactional sex at round 3 reported experiencing sexual violence in the past 12 months compared to 4% of those who did not engage in transactional sex (*p* = 0.024). AGYW who have engaged in transactional sex had more sexual partners in the past 12 months compared to those who did not engage in transactional sex (*p*<0.001). Those who engaged in transactional sex were significantly less likely to have completed Form IV (*p*<0.001) or still be in school and were more likely to reside in a small village (*p* = 0.049).

**Table 1:** Background characteristics for females 14–19 years old in Tanzania at round 3 by engagement in transactional sex

	Mean ± SD or *N* (%)			
	No transactional sex (*N* = 643)	Engaged in transactional sex (*N* = 221)	*N*	*p*‐value^c^
Individual				
Social support index	3.94 ± 0.63	3.92 ± 0.60	864	0.719
Locus of control index	3.30 ± 0.48	3.30 ± 0.44	864	0.868
Self‐esteem index	3.97 ± 0.79	3.86 ± 0.80	864	0.061
Violence sub‐scale (0–6)	3.30 ± 1.81	3.17 ± 1.86	827	0.354
Reproductive health sub‐scale (0–5)^a^	2.58 ± 1.46	2.55 ± 1.39	777	0.822
Sexuality sub‐scale (0–8)^a^	4.61 ± 2.30	4.46 ± 2.33	736	0.415
Decision‐making sub‐scale (0–5)^a^	1.61 ± 1.42	1.53 ± 1.45	860	0.463
Quality of life scale (1–10)	4.99 ± 2.07	4.86 ± 2.03	864	0.426
Age at first sexual intercourse^b^	17.08 ± 1.44	17.19 ± 1.58	280	0.659
Last sex: used condom^b^	18 (31)	100 (45)	280	0.042
Age	15.36 ± 1.35	16.87 ± 1.54	864	<0.001
Experienced sexual violence	11 (3.5)	11 (8.7)	441	0.024
Completed Form IV or still attending school	445 (69)	73 (33)	864	<0.001
Last sex: partner age difference^b^	12 (25)	65 (31)	257	0.405
Has had two or more partners in the past 12 months	5 (0.78)	27 (12)	864	<0.001

Household				
Household size	5.01 ± 1.92	2.08	864	0.573
Head female	416 (65)	147 (67)	864	0.624
Head age	58.41 ± 15.9	60.39 ± 16	864	0.112
Head marital status			864	0.979
Single	9 (1.4)	3 (1.4)	12	
Separated/widowed	414 (64)	144 (65)	558	
Married	220 (34)	74 (33)	294	
Head education			864	0.048
None	262 (41)	110 (50)	372	
Primary	334 (52)	94 (43)	428	
Secondary	47 (7.3)	17 (7.7)	64	

Community				
Village has a market	109 (17)	44 (20)	864	0.320
Distance to market (km)	14.11 ± 15.3	12.85 ± 15.2	864	0.291
Distance to secondary school (km)	4.73 ± 4.58	4.87 ± 4.55	864	0.694
Community gender equitable inheritance scale	3.68 ± 0.699	3.70 ± 0.689	864	0.689
District of residence				
Mufindi	337 (52)	123 (56)	864	0.404
Rungwe	306 (48)	98 (44)	864	0.404
Village size				
Large village	464 (72)	144 (65)	864	0.049
Small village	179 (28)	77 (35)	864	0.049

^a^ Some respondents did not answer certain items and thus sub‐scale numbers vary.

^b^ Asked only to AGYW who had sexually debuted (*n* = 280).

^c^ Estimated using chi‐square tests for categorical variables and standardized *t*‐tests for continuous variables.

### 3.2 Transactional sex and contextual factors (bivariable analysis)

Increasing age and reports of sexual violence were crudely associated with two and three times higher odds of transactional sex, respectively, and having two or more sexual partners in the past 12 months was associated with ∼18‐fold higher odds of transactional sex in the bivariable analyses (Table 2). Current school attendance or completion of Form IV was negatively associated with transactional sex.

**Table 2:** Bivariable associations between sample characteristics and transactional sex (round 3)

	OR	95% CI	*N*
Individual			
Social support index	0.96	0.77–1.19	865
Locus of control index	1.03	0.74–1.43	865
Self‐esteem index	0.84	0.68–1.03	865
Violence sub‐scale (0–6)	0.96	0.88–1.04	828
Reproductive health sub‐scale (0–5)	0.99	0.89–1.10	778
Sexuality sub‐scale (0–8)	0.97	0.91–1.03	736
Decision‐making sub‐scale (0–5)	0.96	0.85–1.08	861
Quality of life scale (1–10)	0.97	0.91–1.04	865
Age at first sexual intercourse	1.04	0.87–1.25	281
Last sex: used condom	1.88	0.91–3.90	
Age	1.94	1.72–2.19**	865
Experienced sexual violence	2.61	1.18–5.79*	442
Completed Form IV or still attending school	0.22	0.15–0.32**	865
Last sex: partner age difference	1.01	0.91–1.12	865
Has had two or more partners in the past 12 months	17.76	6.42–49.09**	865

Household			
Household size	0.98	0.90–1.06	865
Head female	1.08	0.79–1.48	865
Head age	1.01	1.00–1.02	865
Head married	0.97	0.70–1.34	865
Head no education	1.44	1.03‐2.01*	865

Community			
Market in village	1.22	0.81–1.82	865
Mean distance to nearest market (km)	0.99	0.98–1.01	865
Mean distance to secondary school (km)	1.01	0.97–1.05	865
Community gender equitable inheritance scale (0–4)	1.05	0.81–1.36	865
Small village/Mufindi	1.29	0.86–1.94	865
Large village/Mufindi	0.99	0.70–1.40	865
Small village/Rungwe	1.32	0.83–2.09	865
Large village/Rungwe	0.73	0.51–1.03	865

Note: Standard errors adjusted for clustering at the community level. Bivariable analyses run using a crude logistic regression model to estimate odds ratios (OR) and 95% confidence intervals (CI).

*p<0.05; ** *p*<0.01.

### 3.3 Transactional sex and contextual factors (multivariable analysis)

In multivariable analysis, increasing age was associated with nearly two‐fold higher odds of engaging in transactional sex in model 1 (OR = 1.93; 95% CI: [1.67, 2.22]) and model 2 (OR = 1.81; 95% CI: [1.50, 2.18]) and current school attendance or completion of Form IV was associated with decreased odds of transactional sex in model 1 (OR = 0.21; 95% CI: [0.14, 0.31]) and model 2 (OR = 0.23; 95% CI: [0.14, 0.40]; see Table 3). Experiencing sexual violence was not associated with transactional sex at round 3.

**Table 3:** Adjusted associations between contextual factors and transactional sex at round 3

	OR [95% CI]	
	Model 1	Model 2
Age	1.93	1.81
	[1.67–2.22]**	[1.50–2.18]**
Locus of control index	1.05	1.35
	[0.70–1.56]	[0.81–2.23]
Self‐esteem index	0.78	0.87
	[0.62–0.99]*	[0.61–1.22]
Quality of life scale (1–10)	1.02	1.00
	[0.94–1.12]	[0.89–1.12]
Completed Form IV or still attending school	0.21	0.23
	[0.14–0.31]**	[0.14–0.40]**
Experienced sexual violence	–	1.54
	–	[0.53–4.45]
Mean distance to nearest market (km)	0.99	1.00
	[0.97–1.01]	[0.97–1.02]
Mean distance to secondary school (km)	0.97	0.99
	[0.92–1.02]	[0.94–1.05]
Community gender equitable inheritance scale (0–4)	1.08	1.36
	[0.77–1.52]	[0.84–2.20]
Head's highest grade of education: none	1.39	1.14
	[0.91–2.13]	[0.63–2.07]
Head age	1.00	1.00
	[0.99–1.01]	[0.98–1.02]
Head female	1.03	1.08
	[0.71–1.49]	[0.61–1.89]
Small village/Mufindi	1.46	1.51
	[0.82–2.60]	[0.77–2.96]
Small village/Rungwe	1.37	1.39
	[0.69–2.72]	[0.54–3.61]
Large village/Rungwe	0.74	0.92
	[0.42–1.29]	[0.41–2.04]
*N*	864	441

Note: Model 1 is adjusted for age and no education of household head, female‐headed household and district of residence × size of district. Model 2 is adjusted for age and no education of household head, female‐headed household, the experience of sexual violence and district of residence × size of district. Standard errors are adjusted for clustering at the community level.

*p<0.05; ** *p*<0.01.

In qualitative analyses, we found that increasing age for adolescents appeared to be synonymous with more responsibilities even though their economic circumstances remain unchanged. Some of the adolescents suggested that they had hope that their situation of extreme poverty would change by either getting an educational course paid for or getting funds to start a small business. However, for most of these girls, the promises given to them by romantic partners were unfulfilled.

*“I knew I would be at a better place rather than just sitting idle. "I will set business for you can sell even soda, I will set a small shop, even if you don't want soda, you can cook . . . like a restaurant” I said okay, that will be better. But it's just like that, my dream wasn't realised, he conned me. (Female, 17 years, completed Standard 7, Rungwe)*

Further, the interviews revealed that for most adolescents, staying in school is a way of getting out of poverty. Findings show that most adolescents are aware of the risks of engaging in unprotected sex, including unplanned pregnancies. These adolescents, therefore, understood the risk of getting pregnant which could mean dropping out of school.

*My lover convinced me that if I accepted him he would pay for my education and I agreed. He wanted us to have sex but I refused that I couldn't do it while still schooling and told him to wait until I finish my studies. I later accepted and did it; he told me he could enrol me in a tailoring course and that I could continue sewing even when pregnant. He deceived and impregnated me and now I am at home. (Female, 18 years, Rungwe)* .

Qualitative interviews underscored how several females were promised marriage or financial support to attend a school or start a business. However, it appears that due to a situation of financial dependence or when the novelty of the relationship wore off, men left without providing further support, as illustrated by the following quotes:

*I was only 15 years old. Because of hardships, I left here [Mufindi] to go to Dar es Salaam to work. Someone took me there, and it is there that I met one young man. This young man is the one who deceived me, seeing that I had left the village to go to the city; he promised me he would get me out of hardships. (Female, 18 years, Mufindi)*

### 3.4 Impacts of cash plus programme on transactional sex

In the ANCOVA analysis, there was no impact of the intervention on transactional sex at round 3 ([β = 0.003, *p* = 0.905]; Table S3).

## 4 DISCUSSION

This paper examined contextual and individual‐level determinants associated with AGYW engagement in transactional sex and the impact of the *Ujana Salama* “cash plus” programme on transactional sex in rural Tanzania. Our study findings show that increasing age is a risk factor, whereas staying in school was negatively associated with engagement in transactional sex. This is similar to research from rural South Africa by Kilburn et al. (2018) on adolescent schoolgirls (aged 13–20) where the prevalence of transactional sex increased with age until adolescents became young adults (>20 years). Their analysis showed that as adolescent girls transition and age into different stages of life, primary motivations for transactional sex change from resources to fulfil basic needs to a desire to improve their status [9]. In our study, young women also felt the burden of responsibilities increasing as they got older with more pressures around the household and caring responsibilities without an accompanying increase in household or personal resources. Therefore, there is a need to explore the evolving nature of transactional sexual relationships and the multiple pressures young women face as they transition into adulthood.

Staying in school was negatively associated with transactional sex, which is consistent with other research that AGYW who attend and stay enrolled in school have fewer partners and tend to have partners closer to their age compared with young people who do not attend school, hence potentially reducing the risk of sexually transmitted infections (STIs) [40, 41]. Our findings support interventions that encourage girls to attend and stay enrolled in school to promote safer sexual behaviours and also provide educational benefits to improve employment prospects in the future and reduce the need for girls to rely on men to economically provide for them [42, 43, 44].

We also show evidence in the bivariable analysis that sexual violence is associated with increased odds of transactional sex although the results are not statistically significant after adjusting for confounders. There is other research that has shown that engaging in transactional sex, particularly for survival, is often associated with a previous history of sexual victimisation [45]. There is evidence that a traumatic sexual experience affects mental health, with increases in depression or posttraumatic stress disorder, and this may have consequences on sexual risk behaviour. Furthermore, there is some suggestive evidence that experiences of sexual violence have the potential to instil the idea that sex is a commodity to be exchanged for goods or other services, hence perpetuating transactional sexual encounters [46].

Similar to other research in Tanzania [4], previous findings from this “cash plus” study found that the intervention improved economic security via increased business start‐up and livestock tending [47]. However, AGYW are a highly vulnerable population, with few income‐generating opportunities, such as formal employment in the area. AGYW, therefore, report engaging in transactional sex with the hope and intention of increasing their financial independence, but rendering them vulnerable to unsafe sexual behaviours, including falling pregnant and increased childcare responsibilities in tough economic circumstances. Further, as seen in other contexts, gendered unequal power dynamics make it difficult for AGYW who are hoping for an opportunity to change their living circumstances or to obtain necessities, to negotiate for safer sex [6, 48].

Finally, we show that the “plus” intervention had no impact on transactional sex. This may be due to a number of reasons. While this intervention has been previously demonstrated to be protective along several pathway indicators, including gender‐equitable attitudes [49], increased knowledge related to contraception and HIV prevention [50], mental health [51] and economic activities [47], it was perhaps not sufficient to overcome all the interrelated social, relational and economic vulnerabilities which lead AGYW to engage in transactional sex. Alternatively, the lack of impacts may be reflective of alternative drivers of engagement in transactional sex, namely choice of partners, addressing low self‐esteem or a need to boost one's own power and social status [6, 52]. Despite our null result, there is qualitative evidence from similar settings in Tanzania that show that cash transfers can influence transactional sex engagement by altering partner selection criteria from an emphasis on what men could provide to a focus on relationship stability [4]. Our findings show that the mechanisms of impact for a “cash plus” intervention on transactional sex are complex. Economic insecurity is an important driver for engagement in transactional sex, but psychosocial factors and social norms are important and also need consideration in the design of such programmes.

In particular, “plus” interventions addressing transactional sex should target gendered expectations around the male provision and promote concepts of equitable relationships that include critical reflections on agency and power to influence AGYW's choice in partners [29, 30]. Furthermore, programmes that tackle relationship dynamics, individual beliefs and psychosocial aspects of adolescence, alongside structural factors, such as improving economic opportunities for AGYW transitioning to adulthood are promising, especially if tailored to the socio‐economic context to reduce reliance on risky partnerships [31].

Other initiatives in the region have aimed to facilitate safe transitions to adulthood, including reducing HIV incidence. For example, the Determined, Resilient, Empowered, AIDS‐free, Mentored and Safe (DREAMS) Partnership combines structural, behavioural and biomedical interventions to reduce HIV incidence among AGYW. While interventions vary across sites, the evidence suggests that DREAMS interventions reduced unsafe sex without condoms, violence from partners, and the number of sex clients, and improved the uptake of pre‐exposure prophylaxis (PrEP), condom use negotiation and knowledge of partner's HIV status in Zimbabwe [53]. Further, qualitative data from Tanzania suggest that DREAMS cash transfers may have reduced AGYW engagement in transactional sex for basic needs; however, this was not confirmed quantitatively [31]. Nevertheless, DREAMS has not demonstrated evidence of reduced HIV incidence in Zimbabwe [53]. Further population‐based studies of HIV prevalence in South Africa and Kenya indicated substantial declines in HIV incidence among AGYW, but these trends began before DREAMS was implemented and did not accelerate during DREAMS’ implementation [54]. Additionally, many DREAMS implementers are NGOs, limiting the scalability and sustainability of the interventions once funding from the DREAMS initiative ends, and the evaluations are either non‐randomised or qualitative in nature, which limits the ability to identify causal impacts. Thus, the added value of our study is a sustainable and scalable approach, as Ujana Salama is owned by Government and implemented through its existing structures, with a gold standard—RCT—evaluation design.

Interventions should also focus on increasing school enrolment and attainment, particularly among older adolescent girls or young women. For instance, interventions that are aimed at keeping girls in school are critical to promoting safe sexual behaviours and preventing STIs. However, effectively preventing infections in young women should also involve the development of interventions for young women out of school or who have completed school that encourages them to be part of safer sexual networks. Despite these potential benefits, a focus on programmes that aim to address individual‐level knowledge and behaviours without addressing structural inequalities will only go so far in reducing risk and promoting safe transitions to adulthood. Thus, policy change and broader enabling environments, promoting gender equality, stronger health systems, and more inclusive economic opportunities are needed.

This study had some strengths and limitations. As this study was a large RCT, the data were subject to rigorous quality checks. We also had a large sample size and used a measure of transactional sex that was cognitively tested in Tanzania and other east African countries to measure the primary motivation for transactional sex in order to assess young women's risk when engaging in transactional sex. For instance, when financial motivation is the main reason, women may find it hard to negotiate condom use due to the material nature of the negotiation, making it risky for STIs. However, we acknowledge the limitation of social desirability that plays an important role in self‐reported sexual behaviours and that the expected direction of social desirability bias is that respondents will over‐report condom use and under‐report the number of sexual partners [55]. Even the questions on transactional sex may be under‐reported as, unlike female sex workers who self‐identify as sex workers, young women engaging in transactional sex seldom disclose that they have exchanged sex for money [8]. Despite efforts implemented to reduce social desirability bias, such as building rapport and training enumerators on sensitive topics and ethics, it is important to acknowledge the role that social desirability bias might play in the interpretation of findings. Furthermore, low rates of transactional sex may limit our statistical power to detect associations with determinants of interest. Finally, the youth in this study come from extremely poor households in a rural setting, and findings may not generalise to adolescents from higher socio‐economic households and households in urban settings.

## 5 CONCLUSIONS

Given its complex determinants, programmes aimed at reducing transactional sex need to address risk factors driven by both social and structural environments. Existing research has not examined many interventions that have successfully reduced transactional sex, and while we did not find protective impacts of the cash plus programme, we did find that schooling was associated with lower odds of transactional sex, and that sexual violence and transactional sex can be mutual reinforcing risk factors. Thus, combination prevention interventions that aim to complementarily address structural drivers of HIV infection should focus on barriers to school attendance and economic vulnerability, alongside aims, such as improving self‐esteem or cultivating aspirations and addressing gendered social norms.

## COMPETING INTERESTS

The authors declare no competing interests.

## AUTHORS’ CONTRIBUTIONS

MR, TP, LK, SQ and UG designed the study. SQ conducted the statistical analyses and contributed to drafting the manuscript. TP, SQ and MR interpreted statistical analyses. LK led the qualitative analyses. All authors contributed to the interpretation of findings, drafting of the manuscript and approved the final submitted version. Members of the Tanzania Adolescent Cash Plus Evaluation team further contributed to the study design and data collection.

## FUNDING

Funding for this pilot and evaluation has been provided by Oak Foundation (#OCAY‐16‐73) and UNICEF. Additional funding for the evaluation (2017–2019) was provided by the UK's Department of International Development (DFID 203529‐102) and the Swedish Development Cooperation Agency (Sida G41102), both through a grant to UNICEF Office of Research—Innocenti supporting the Transfer Project. Additional funding for program implementation activities (2018–2020) was provided by Irish Aid (Irish Aid IA‐TAN/2019/064). The funders had no role in the analysis or interpretation of data.

## Supporting information


**Figure S1**: Cash plus timeline.

**Table S1**: Topics in the training curriculum.

**Table S2**: Variable definitions at the individual, household and community level.

**Table S3**: Program impacts on transactional sex at round 3, Analysis of covariance (ANCOVA).

**File S1**: Members of the Tanzania Cash Plus Evaluation Team.

## Data Availability

Data from this study are not currently publicly available but expect to be made available by 2024, pending approval from UNICEF and the Government of the Republic of Tanzania.
