# Supplementary material for: Transactional sex among adolescent girls and young women enrolled in a cash plus intervention in rural Tanzania: a mixed‐methods study
Source: J Int AIDS Soc. 2022 Nov 30;25(12):e26038. doi: 10.1002/jia2.26038 (PMC9712808; doi:10.1002/jia2.26038)
Supplement: Supplementary file 1 — Figure S1: Cash plus timeline. [file JIA2-25-e26038-s003.docx]

**Figure S 1: Cash plus timeline**
