# Supplementary material for: Transactional sex among adolescent girls and young women enrolled in a cash plus intervention in rural Tanzania: a mixed‐methods study
Source: J Int AIDS Soc. 2022 Nov 30;25(12):e26038. doi: 10.1002/jia2.26038 (PMC9712808; doi:10.1002/jia2.26038)
Supplement: Supplementary file 2 — Table S1: Topics in the training curriculum. [file JIA2-25-e26038-s001.docx]

**Table S1. Topics in the training curriculum**

| **Session** | **Livelihood skills** | **Reproductive Health** |
| --- | --- | --- |
| **Week 1** | **Transformation**   - A concept of transformation - Five stages of human transformation | **Coping with Puberty**   - Menstruation - Wet dreams - Coping with community expectations at and after puberty |
| **Week 2** | **Dream**   - Living well with surrounding community - 5 types of personalities in the community | **Relationships**   - What makes a good friend: Boyfriends/girlfriends: - Love, sex and consent; risk perception with regard to SRH - Decision making - Assertiveness, negotiating skills and body language |
| **Week 3** | **Business Concepts**   - Developing business ideas/ self-evaluation/ - Success factors in personal economic development | **HIV and AIDS**   - Prevention and protection - What do I know about HIV: the HIV wall, Q and A - Condoms are forever |
| **Week 4** | **Generate your business idea**   - Types of people in the community/ learning from role models in the community | **Sexual Risk Taking and Protection**   - Risk taking - Major sexual risks and why they are risks |
| **Week 5** | **SWOT analysis**   - Ranking and prioritization | **Violence and GBV**   - What is it - How to address it |
| **Week 6** | **Developing simple business plan**   - Filling simple business plan forms | **Consequences of risk-taking: pregnancy,**   - What to do if pregnant/partner pregnant: - Protecting the baby: ante and post-natal care: - Abortion, the law, unsafe abortion |
| **Week 7** | **Record keeping**   - Understanding simple business record keeping. | **Family planning**   - Most common methods used, advantages and disadvantages: Accessing family planning services in and around the community - Why condoms: double protection |
| **Week 8** | **Saving for business**   - Establishing micro savings and loan groups | **Sexually Transmitted Infections (STIs)**   - Consequences of risk taking: STIs - The most common STIs, symptoms and treatment Relationship between STIs and HIV: Stigma: fear and morality |
| **Week 9** | **Responsibilities of an entrepreneur**   - Legal aspects of business, insurance and licenses | **Living with HIV**   - Testing for HIV and disclosure - Living with HIV: ART treatment, nutrition, avoiding re-infection, having children and PMTCT - AIDS, and opportunistic infections especially TB |
| **Week 10** | **Long term life/business plans**   - Setting carrier goals and long-term business plans. Identify linkage and referral pathways | **Alcohol and Drug abuse**   - Prevalence of alcohol and drugs among adolescents and why - The impact of alcohol and drugs - Resisting peer/partner pressure |
