# Supplementary material for: Transactional sex among adolescent girls and young women enrolled in a cash plus intervention in rural Tanzania: a mixed‐methods study
Source: J Int AIDS Soc. 2022 Nov 30;25(12):e26038. doi: 10.1002/jia2.26038 (PMC9712808; doi:10.1002/jia2.26038)
Supplement: Supplementary file 3 — Table S2: Variable definitions at the individual, household and community level. [file JIA2-25-e26038-s002.docx]

**Table S2: Variable definitions at the individual, household and community level**

| **Variable** | **Type** | **Range** | **Definition** |
| --- | --- | --- | --- |
|  | **Individual** | | |
| Age (at baseline) | Continuous | 14-19 | Age in years reported by youth. |
| Age at first sex | Continuous | 12-22 | Among those who reported having sex, respondents were asked to report the age of their first sexual experience. |
| Social support index | Continuous | 1-5 | Based on the average score (range: 1-5) across four items measuring adolescents’ perceived social support. A higher score corresponds to greater perceived support available to the adolescent. Possible responses to the following prompts range from 1 ‘strongly disagree’ to 5 ‘strongly agree: a) *I have friends with whom I can share my joys and sorrows;* b) *I can talk about my problems with my friends;* c) *I get the help and support I need from my family;* d) *My family is willing to help me make decisions.* |
| Locus of control | Continuous | 0-5 | Based on average score (range: 1-5) across five items of Levenson's locus of control scale (35). A higher score corresponds to greater sense of internal control. Possible responses to the following prompts range from 1 'strongly disagree' to 5 'strongly agree': a) *It's not always wise for you to plan too far ahead because many things turn out to be a matter of good or bad fortune.* b) *Your life is determined by your own actions.* c) *When you get what you want, it is usually the result of your own actions.* d) *You feel like what happens in your life is mostly determined by others.* e) *Getting what you want requires pleasing the influential people.* |
| Self-esteem index | Continuous | 0-5 | Based on the average score (range: 1-5) across two items of Rosenberg's self-esteem scale questions (36). Higher scores correspond to higher self-esteem. Answers range from 1 'strongly disagree' to 5 'strongly agree': a) *You feel that you have many good qualities.* b) *On the whole, you are satisfied with yourself.* |
| Gender Equitable Men (GEM) Violence sub-scale | Continuous | 0-6 | A sub-scale developed using the violence-related questions of the modified GEM scale (range: 0-24) administered to adolescents (37, 38). Higher scores indicate more equitable attitudes. Response options included ‘agree’; ‘partially agree’; or ‘do not agree’ for the following prompts: a) *There are times a woman deserves to be beaten;* b) *A woman should tolerate violence in order to keep her family together;* c) *If someone insults a man he should defend his reputation with force if he has to;* d) *It is okay for a man to hit his wife if she will not have sex with him;* e) *A man using violence against his wife is a private matter that should not be discussed outside the couple;* and f) *It is alright for a man to beat his wife if she is unfaithful.* We code ‘agree’ of ‘partially agree’ responses to equal zero and ‘do not agree’ to equal 1 and then generate a sum of these indicators. |
| Reproductive Health sub-scale | Continuous | 0-5 | A sub-scale developed using the reproductive health-related questions of the modified GEM scale (range: 0-24) administered to adolescents (37, 38). Higher scores indicate more equitable attitudes. Response options included ‘agree’; ‘partially agree’; or ‘do not agree’ for the following prompts: a) *It is a woman’s responsibility to avoid getting pregnant;* b) *A man should be angered/shocked if his wife asks him to use a condom;* c) *Women who carry condoms on them are easy;* d) *Only when a woman has a child is she a real woman;* and e) *A real man produces a male child.* We code ‘agree’ of ‘partially agree’ responses to equal zero and ‘do not agree’ to equal 1 and then generate a sum of these indicators. |
| Sexuality sub-scale | Continuous | 0-8 | A sub-scale developed using the sexuality-related questions of the modified GEM scale (range: 0-24) administered to adolescents (37, 38). Higher scores indicate more equitable attitudes. Response options included ‘agree’; ‘partially agree’; or ‘do not agree’ for the following prompts: a) *It disgusts me when I see a man acting like a woman;* b) *A woman should not initiate sex;* c) *You do not talk about sex, you just do it;* d) *A woman who has sex before she marries does not deserve respect;* e) *Men need sex more than women do;* f) *Men are always ready to have sex;* g) *A man needs other women, even if things with his wife are fine;* and h) *It is the man who decides how he wants to have sex.* We code ‘agree’ of ‘partially agree’ responses to equal zero and ‘do not agree’ to equal 1 and then generate a sum of these indicators. |
| Household decision making sub-scale | Continuous | 0-5 | A sub-scale developed using the household decision making-related questions of the modified GEM scale (range: 0-24) administered to adolescents (37, 38). Higher scores indicate more equitable attitudes. Response options included ‘agree’; ‘partially agree’; or ‘do not agree’ for the following prompts: a) *Giving the kids a bath and feeding them are the mother’s responsibility;* b) *A woman’s most important role is to take care of her home and cook for her family;* c) *A man should have the final word on decisions in his home;* d) *The husband should decide what major household items to buy;* and e) *A woman should obey her husband in all things.* We code ‘agree’ of ‘partially agree’ responses to equal zero and ‘do not agree’ to equal 1 and then generate a sum of these indicators. |
| Quality of life | Continuous | 1 - 10 | Youth quality of life was self-reported with scores based on their responses to the following prompt: *Imagine a ladder where on the bottom, the first step, represents the worst possible life for you and the highest step, the tenth, represents the best possible life for you. On which step of the ladder would you say you are today?* |
| School attendance or competed Form IV | Binary | 0: No 1: Yes | Based on youth responses to whether they are currently attending school or if they have completed Form IV. |
| Sexual violence | Binary | 0: No 1: Yes | Youth were considered to have experienced sexual violence in the past 12 months if they responded 'sometimes' or 'often' to a) *How often have you been forced to have sexual intercourse or to perform other sexual acts against your will?* OR an affirmative response to b) *In the past 12 months, has anyone ever touched you in a sexual way without your permission, but did not try and force you to have sex?*. For ethical reasons, this indicator was collected via split-sample approach and is thus available for only half of the sample. |
| Age difference between respondent and partner at last sex | Continuous | -4-14 | Sexually debuted respondents are asked to provide the age of their last sexual partner. From that, we calculated the difference between the respondent’s age and the partner’s age at that time. |
| Last sexual partner is more than 5 years older | Binary | 0: No 1: Yes | Using the age difference indicator above, we calculate whether the respondent’s partner was more than 5 years older at last sex. |
| Number of sexual partners in past 12 months | Binary | 0: 1 or less  1: 2 or more | Youth were asked the number of sexual partners they had in the past 12 months (if they reported sexual debut). We then dichotomized this variable as equal to 0 if they reported less than 2 partners and equal to 1 if they reported two or more partners. Those who did not sexually debut were coded as 0 for this variable. |
|  |  | **Household** | |
| Household size | Continuous | 1 - 14 | Reported by the household head. |
| Female headed household | Binary | 0: No 1: Yes | Reported by the household head. |
| Age of household head | Continuous | 18-109 years | Reported by the household head. |
| Household head marital status: married | Categorical | 0: Single 1: Separated/Widowed/Married | Reported by the household head. Response options include: a) *Single/never married;* b) *Divorced;* c) *Separated;* d) *Widowed;* e) *Customary marriage, monogamous;* f) *Customary marriage, polygamous;* g) *Religious marriage, monogamous;* h) *Religious marriage; polygamous;* i) *Cohabitating, monogamous;* j) *Cohabitating, polygamous;* k) *Civil marriage.* For regression analyses, we use a dummy variable equal to one if the head is married and equal to zero otherwise. *For descriptive analysis in Table 1, we include three variables, indicating whether the head was Single/never married (=1 if yes and =0 otherwise); Divorced, separated, or widowed (=1 if yes and =0 otherwise); and married (=1 if yes and =0 otherwise)*. |
| Household head educational attainment: none | Categorical | 0: Head as some formal education 1: Head has no formal education | Reported by the household head. The head was asked “*Did NAME ever attend school?”* If the head responded “*No”*, we coded educational attainment: none equal to 1, if the head responded “*Yes”*, they were then asked *“What was the highest class completed?”* Response options included: a) *Pre-Primary;* b) *Standard I,* c) *Standard II;* d) *Standard III;* e) *Standard IV;* f) *Standard V;* g) *Standard VI;* h) *Standard VII;* i) *Primary + Course;* j) *Form I;* k) *Form II;* l) *Form III;* m) *Form IV;* n) *Form IV + Course;* o) *Form V;* p) *Form VI;* q) *Form VI + Course;* r) *Ordinary Diploma;* s) *University I;* t) *University II;* u) *University III;* v) *University IV +;* w) *University V +;* x) *Adult/Vocational Training.* If responses indicated individuals had any formal education (a-x), we coded to 0 for regression analyses. For descriptive analysis in Table 1, we include three categories, separating out those who have *Pre-Primary* and *Standard VII as “primary” and all others as “secondary or above.”* |
|  |  | **Community** | |
| Market in village | Binary | 0: No 1: Yes | A knowledgeable community member was asked *“Is there a daily market in this village?”* and *“Is there a larger weekly market in this village?”* A *“Yes”* response to either of those questions was coded as equal to 1 and 0 otherwise. |
| Distance to market | Continuous | 0-72 km | Reported by knowledgeable community member. Asked to report the distance to the nearest daily or weekly market in the village in kilometers or miles. |
| Distance to secondary school | Continuous | 0-30 km | Reported by knowledgeable community member. Asked to report the distance to the nearest secondary school market in the village in kilometers or miles. |
| Community gender equitable inheritance scale | Continuous | 0-4 | Based on the score calculated by adding the responses (0: No, 1: Yes) to the following: *When a husband dies, the wife can inherit* a) *land*, b) *the house*, c) *other property*, and d) *The widow can be inherited by a brother or other male* (0: Yes, 1: No). A higher scale value indicates more gender equitable inheritance norms in the community. Assessed using the community questionnaire administered to a knowledgeable community member. |
