# Supplementary material for: Transactional sex among adolescent girls and young women enrolled in a cash plus intervention in rural Tanzania: a mixed‐methods study
Source: J Int AIDS Soc. 2022 Nov 30;25(12):e26038. doi: 10.1002/jia2.26038 (PMC9712808; doi:10.1002/jia2.26038)
Supplement: Supplementary file 4 — Table S3: Program impacts on transactional sex at round 3, Analysis of covariance (ANCOVA). [file JIA2-25-e26038-s004.docx]

**Table S3. Program impacts on transactional sex at round 3, Analysis of covariance (ANCOVA)**

|  | **Transactional sex**  **Coefficient (SE)** |
| --- | --- |
| Baseline transactional sex experience | 0.38** |
|  | (8.30) |
| Treatment (Cash Plus village) | 0.00 |
|  | (0.07) |
| District/ Village size (ref= Mufindi/large village) |  |
| Mufindi/small village | 0.04 |
|  | (1.00) |
| Rungwe/small village | 0.05 |
|  | (1.31) |
| Rungwe/large village | -0.02 |
|  | (0.70) |
| Age | 0.09** |
|  | (9.92) |
| *N* | 864 |

* *p*<0.05; ** *p*<0.01. Model is adjusted for baseline experiences of transactional sex, youth age at baseline, district of residence, and size of district of residence.
