# Supplementary material for: Transactional sex among adolescent girls and young women enrolled in a cash plus intervention in rural Tanzania: a mixed‐methods study
Source: J Int AIDS Soc. 2022 Nov 30;25(12):e26038. doi: 10.1002/jia2.26038 (PMC9712808; doi:10.1002/jia2.26038)
Supplement: Supplementary file 5 — File S1: Members of the Tanzania Cash Plus Evaluation Team. [file JIA2-25-e26038-s005.docx]

Members of the Evaluation Team include: University at Buffalo: Tia Palermo (co-principal investigator), Sarah Quinones; UNICEF Office of Research: Lusajo Kajula, Jacobus de Hoop, Leah Prencipe, Valeria Groppo, Nyasha Tirivayi and Jennifer Waidler; EDI Global: Johanna Choumert Nkolo (co-principal investigator), Respichius Mitti (co-principal investigator), Marie Mallet and Bhoke Munanka; TASAF: Paul Luchemba and Tumpe Mnyawami Lukongo; TACAIDS: AroldiaMulokozi; UNICEF Tanzania: Ulrike Gilbert, Paul Quarles van Ufford, Rikke Le Kirkegaard, Frank Eetaama, JenniferMatafu, Diego Angemi, Luisa Natali.
